# The Effect of Music Therapy on Cognitive Functioning Among Older Adults

**DOI:** 10.1093/geroni/igaa057.2837

**Published:** 2020-12-16

**Authors:** Kuei-Min Chen

**Affiliations:** Kaohsiung Medical University, Kaohsiung City, Taiwan (Republic of China)

## Abstract

Music creates and fosters connection and interrelationships between individuals and encourages social interaction. Indeed, community-based interventions are a powerful way to engage older people. This presentation will provide an overview of music therapy and the impact it can have on the cognitive functioning of older people. Examples of music therapy interventions in the community can be found in nursing homes, hospitals, or communities. Differences in receptive (passive) music therapy and active music therapy will be discussed along with the impact these therapies may have on individuals experiencing cognitive decline. Moreover, studies of music therapy combined with other activities, such as exercise or art will be discussed. This research will be presented within the context of the recommendations put forth by the Global Council on Brain Health aimed at adults aged 50+.

